# Emerging multi-drug resistant and extended-spectrum β-lactamase (ESBL)-positive enterotoxigenic *E. coli* (ETEC) clones circulating in aquatic environments and in patients

**DOI:** 10.1016/j.onehlt.2025.100968

**Published:** 2025-01-10

**Authors:** Enrique Joffré, Alberto J. Martín-Rodríguez, Annie Justh de Neczpal, Astrid von Mentzer, Åsa Sjöling

**Affiliations:** a Department of Chemistry and Molecular Biology, University of Gothenburg, Gothenburg, Sweden; b Department of Microbiology and Tumor Biology, Karolinska Institutet, Stockholm, Sweden; c Department of Clinical Sciences, Universtiy of Las Palmas de Gran Canaria, Las Palmas de Gran Canaria, Spain; d Department of Microbiology and Immunology, University of Gothenburg, Gothenburg, Sweden

## Abstract

Diarrheal disease pathogens often spread through water-borne routes. Enterotoxigenic *Escherichia coli* (ETEC) is a major bacterial agent causing diarrheal disease in children, adults, and travelers in endemic areas. In addition, ETEC is responsible for outbreaks of water and food-borne gasteroenteritis globally, ETEC isolates also show robust survival capacity in various environmental settings, including aquatic environments.

During the last decade, studies of ETEC isolates have indicated a rapid increase in multi-drug resistant and extended-spectrum β-lactamase (ESBL)-positive human-specific ETEC strains. These have been found in both environmental water sources and human patients, warranting the urgent need for focused monitoring of antibiotic resistance development in ETEC.

Whole genome sequencing (WGS) of isolates from environmental, animal, and human sources enables in silico surveillance of emerging pathogenic and multi-drug resistant strains. This method allows for re-analysis of genomic data, aiding in identification of new variants of pathogenic clones.

By integrating data from diverse sources inclusing sequenced isolates, we found that certain ETEC clonal lineages e.g., those expressing certain toxin-colonization factor profiles including STp/CS6, LT STh/CS2 + CS3, and LT STh/CFA/I are more at risk to develop multi-drug resistance than other ETEC lineages. Comparizon of multi-locus sequence types from papers with WGS data indicated ST182, ST4, ST2332 and new ST types to be emerging multi-drug resistant ETEC.

We conclude that further studies on sequenced ETEC/*E. coli* genomes are needed to enhance our understanding of the dynamics of ETEC evolution, and the relation of virulence and resistance profiles in both environmental and clinical isolates.

## Graphical abstract

**Figure:**
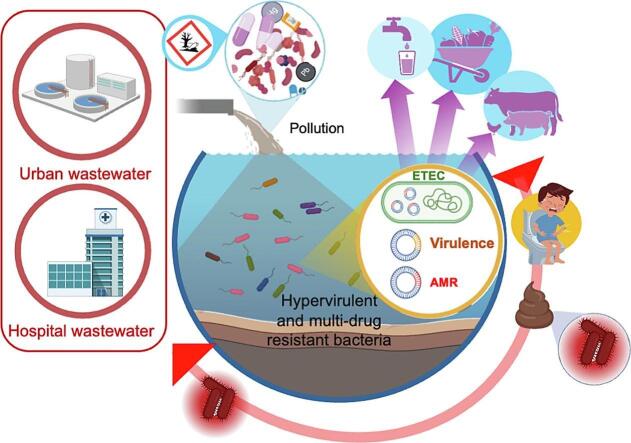

## 1 Diarrheal diseases and enterotoxigenic *Escherichia coli* (ETEC)

Diarrheal disease remains a major cause of morbidity and mortality in children globally being the second leading cause of death with 370.000 deaths yearly in children under five years old [www.who.int]. Even though mortality has declined over the last decades, morbidity remains high, with severe impact on the immune system and leading to malnourishment, stunting, and impaired cognitive development after repeated episodes of diarrhea [www.who.int]. Two landmark global studies on childhood diarrhea, the Global Enteric Multicenter Study (GEMS) [1] and Etiology, Risk Factors and Interactions of Enteric Infections and Malnutrition and the Consequences for Child Health and Development study, abbreviated as MAL-ED [2,3] identified *Shigella* spp., *Campylobacter* spp., enteropathogenic *E. coli* (EPEC), and enterotoxigenic *E. coli* (ETEC) as the primary bacterial pathogens responsible for childhood diarrheas. Subsequent re-examination of these results using molecular methods indicated that ETEC and *Shigella* spp. account for even more causes than previously estimated [4]. Consequently, there is a growing emphasis on the development of vaccines as preventive measures towards the severe secondary effects caused by these pathogens, especially in young children [5].

ETEC is also considered the most common cause of traveler's diarrhea [6]. In Western countries, ETEC is responsible for sporadic food-borne outbreaks of gastroenteritis, causing an estimated 10 million cases annually [7]. Additionally, there is an increased risk of developing irritable bowel syndrome (IBS) and/or persistent abdominal symptoms following an ETEC infection [8].

Like many other enteropathogens, ETEC is transmitted through contaminated food and water [9]. Upon ingestion, it causes infection in the ileum of the distal small intestine, where colonization is mediated by plasmid-encoded colonization factors (CFs). CFs constitute a heterogenous group of fimbrial, fibrillar and afimbrial structures on the bacterial surface that adhere to human enterocytes. At present ca 30 different CFs have been described [10]. CFs may be expressed alone or in various combinations by different ETEC strains and/or clonal lineages, with the most prevalent being CFA/I, CS1-CS6 and CS21 [[10], [11], [12]].

After adhering to the epithelium in the ileum, ETEC cause massive loss of water and electrolytes by means of one or two secreted enterotoxin types: the heat-labile (LT) and heat-stable (STp and/or STh) toxins [11]. Both toxins induce intracellular signaling, leading to dysregulation and opening of ion channels in the apical membrane of intestinal enterocytes causing fluid loss. The carriage of these toxin genes is the hallmark of ETEC as a pathogen [[11], [12], [13]], it is however important to note that heat-labile and heat stable toxin variants as well as animal specific CFs are present in ETEC causing porcine and bovine diarrhea [11], which is not covered in this review. In human ETEC isolates certain toxin/CF combinations are associated with other virulence genes such as *etpBAC*, *eatA* and *cexE,* which has been extensively reviewed in a previous publication [13].

## 2 Antibiotic resistance in ETEC

Infection with ETEC typically manifests as acute watery diarrhea, accompanied by stomach cramps, vomiting and less frequently fever, with symptoms lasting between 1 and 3 days [[6], [7], [8], [9], [10], [11], [12], [13], [14]]. While the recommended treatment for ETEC diarrhea is oral or intravenous rehydration, which is generally effective if administered promptly [11,15], antibiotic use has become increasingly common in managing moderate to severe infections [6]. This includes not only the treatment of active infections but also the preventive administration of antibiotics to travelers, a practice prevalent in Europe and even more so in U.S.A [16]. Additionally, self-medication with potentially inappropriate antibiotics is widespread [15].

Historic data shows that ETEC strains isolated before 1990 carried resistance genes towards ampicillin (*ampC*) and occasionally sulfonamides (*sul1/2*), streptomycin (*strAB*), trimethoprim (*dhfr)*, and tetracycline (*tetA*) [17]. However, recent publications indicate that certain clonal lineages of ETEC have recently developed multi-drug resistance, including extended-spectrum β-lactamases (ESBL), fluoroquinolone resistance (*qnr*) and azithromycin resistance [[18], [19], [20]].

Azithromycin (AZM), ciprofloxacin (CIP), and rifaximin are often prescribed for treating and/or preventing traveler's diarrhea [15,21]. The increased presence of antibiotic resistance genes (ARGs) towards these antibiotics in ETEC probably reflects extensive use. Ouyang-Latimer et al. reported a substantial increase in resistance to CIP and AZM when comparing isolates collected in 1997 with isolates collected between 2006 and 2008 among EAEC and ETEC isolates from travelers to Mexico, Guatemala, and India [22]. Guiral et al. found that CIP and cefotaxime (CTX) resistance have increased in the last decade in ETEC, particularly in isolates from travelers to South Asia/India [23]. A study in Nepal found that ESBL postive ETEC increased to 30 % after 2013 but was barely detected in isolates collected before 2013 [18]**.** Hence it is evident that the use of antibiotics as a preventive treatment exposes infectious ETEC isolates to antibiotics and thereby increase the risk of development of multi-drug resistance.

Authors of this review performed a study of whole genome sequenced (WGS) ETEC isolates isolated between 1980 and 2011 from 30 different countries [12]. The results showed that ETEC consists of clonal lineages with defined toxin and CF profiles [12]. The prevalence of antibiotic resistance (ABR) was not reported in that study, but re-analysis of the data indicate *ampC* and occasionally *sul1/2*, *strAB*, *tetA,* and *dhfr* ARGs while the levels of *qnrS* were low (8 %) and ESBL genes were completely absent from this dataset. However, recent publications indicate that multi-drug resistance including ESBL carriage is increasing in ETEC [18,20,23]. Sequencing of plasmids revealed that ARGs often come in gene cassettes inserted into specific plasmids in ETEC [20,24,25].

## 3 Emergence of the ESBL pandemic in *E. coli*

Antibiotic resistant Enterobacterales are prevalent across all countries and continents [26]; however, the problem might be more severe in low-and-middle income countries. For instance, a recent study in Dhaka, Bangladesh between 2015 and 2019 showed that 97 % of *E. coli* were multi-drug resistant [27]. Low-and-middle-income countries might contribute to the spread and emergence of new resistant pathogenic isolates, not only because of high prevalence of infectious diseases and reduced access to clean drinking water, but also since prescription of antibiotics is less regulated and the absence of, or underdeveloped, waste-water treatment plants (WWTPs) [28].

ESBL-producing *E. coli* are increasing and constitute an emerging global health problem. The widespread use of antibiotics in both human and veterinary medicine, as well as preventive or growth-promoting use in animal husbandry, has led to the global spread of multi-drug resistant bacteria. Certain clones of *E. coli* frequently carry ESBL genes and the same clones or lineages are often detected in aquatic sources and in WWTPs. The most infamous among these is multi-locus sequence type (MLST) ST131, a clone encompassing *E. coli* that commonly cause urinary tract infections and sepsis [29]. The continued rapid increase of ESBL producing ST131 sequence types has led to what is now referred to as the ‘CTX-M pandemic’ as ST131 preferably carries the ESBL gene *bla*_CTX-M-15_. CTX-M-15 confers resistance to cephalosporins e.g., cefotaxime (CTX), ceftriaxone (AXO) and ceftazidime (CAZ), and is the most common ESBL variant globally**.** ST131 isolates are often isolated from hospital sewage and WWTPs likely due to the substantial bacterial load shed in urine and feces from infected individuals, combined with its apparent ability to persist in waste-water environments [30]. In studies on ETEC isolates from Nepal *bla*_CTX-M-15_ was detected in 80 % of the ESBL positive isolates [18] and *bla*_CTX-M-15_ was found in ETEC isolates from airplane waste together with *qnrS1, dfrA17*, *aadA5*, *qacE*Δ*1*, *sul1*, and *mphA.* These studies indicate that *bla*_CTX-M-15_, often in combination with multi-drug resistance [18,20,23], is the most common ESBL gene in ETEC as well.

## 4 Spread of resistance genes by mobile genetic elements in aquatic environments

The dissemination of ARGs often occurs via mobile genetic elements (MGEs), including plasmids and integrative and conjugative elements (ICEs), which facilitate their transfer within and between species [31]. Plasmids often carry cassettes of resistance genes and insertion sequence (IS) elements, enabling horizontal transfer to a new bacterial host [32,33]. Plasmid-borne transmission is believed to be the cause of the rise of carbapenem-resistance and ESBLs in Enterobacterales, as well as the recent emergence of colistin resistance on IncX4 and IncI2 plasmids [34]. Despite the potential fitness cost of plasmids to host bacteria, they are maintained in bacterial populations even in the absence of antibiotic selective pressure. Hence, genes transferred by plasmids and ICEs may confer other advantages beyond antibiotic resistance, for example by aiding bacterial survival in harsh environments outside the host [[35], [36], [37]]. Traits conferred by plasmidborne genes include enhanced biofilm formation [38,39], aggregation [40], metabolic plasticity [41], resistance to UV and heat [42], competition [43], and resistance to chemicals [44,45].

ESBL-producing *E. coli* are often multi-drug resistant, carrying an arsenal of other ARGs on plasmids or other MGEs, or integrated in the chromosome. IncF plasmids, commonly found in *E. coli* from human, animal and environmental water sources, are typically carriers of ESBL genes [46,47]. Several studies report that IncFII plasmids play a key role in the global spread of *bla*_CTX-M-15_ in human ST131 and ST405 *E. coli* clones [37,48,49]. The *bla*_CTX-M-15_ gene can also be present in other plasmid Inc. types, including IncN [44,50], IncL/M [51], IncHI2 [52], IncX4 [53] and IncI1 [54].

## 5 Water, wastewater and hospital sewage as sources of multi-drug resistant ETEC

ETEC is considered a waterborne pathogen and several studies have identified ETEC in river and waters [[55], [56], [57]]. Prolonged survival of ETEC in water, an oligotrophic environment, for up to three months in seawater and freshwater has been documented [58,59]. This survival is associated with transcriptional alterations in lipopolysaccharide and carbohydrate modification pathways [60]. It is thought that water ecosystems may represent not only a transient route of transmission but also a natural reservoir for ETEC [9]. The waterborne transmission is emphasized by the link between epidemic outbreaks of ETEC and other diarrheal pathogens during flooding [11,61].

Wastewater transmission of ABR bacteria is recognized as an emerging threat. WWTPs are considered hotspots for emergence and dissemination of antibiotic resistant bacteria since they receive sewage from different sources such as households, hospitals, land run-offs and industries often mixed with high levels of contaminants such as antibiotic residues [33,[62], [63], [64], [65]]. Given that up to 10^9^ bacteria/ml are shed in diarrheal stool [24], and an infected individual can lose several liters of stool per day, the potential for widespread transmission of emerging diarreal multi-drug resistant *E. coli* through fecal accumulation in WWTPs is considerable. ARGs are most concentrated in inlets of WWTPs, therefore, WWTPs can be used to monitor prevalence of pathogens that acquired ARGs at a community scale.

Despite their high efficiency, conventional WWTPs are unable to completely remove bacteria, a problem that is exacerbated by the rising proportion of multi-drug resistant bacteria in the effluents, thereby reintroducing hazardous strains into the environment upon outlet discharge [66]. A surveillance study of hospital wastewater, community sewage and the receiving urban WWTP found the prevalence of ESBL *E. coli* to be 11.55 %, 6.9 % and 3.7 % respectively [30]. A 1-year study in Sweden analysing urban wastewater and hospital wastewater found 2.4 % of the *E. coli* isolates in the urban effluents and 14.0 % of the isolates from the hospital effluent to be resistant to all tested beta-lactam antibiotics, with 97 % of these confirmed to be ESBL producers [67].

Higher detection frequency of ESBL-producing and multi-drug resistant bacteria in treated effluents compared to untreated wastewater has previously been reported [68]. These results suggest that certain multi-drug resistant *E. coli* can survive, or even thrive, through the WWTP process. This hypothesis of bacterial persistence and potential selection in wastewater has been proposed by some studies [30,68]. ETEC has been shown to persist in wastewater and in treated effluents in endemic areas [69,70]. However, whether ESBL-producing ETEC have increased fitness in wastewater still requires confirmation.

The problems associated with the presence of ETEC (and other bacterial pathogens) in treated wastewater effluents is multifactorial, and involves reintroduction of pathogens in the environment upon discharge of outlet water where sub-lethal concentrations of antibiotic and antibiotic residues may occur. Altogether, this highlights the need for integrated surveillance efforts considering all the environmental components beyond the water column, as well as research efforts devoted to the understanding of *E. coli* adaptation to environmental niches outside a living host.

## 6 ETEC multi-locus sequence types, MLST, associated with multi-drug resistance carriage

ETEC remains a significant global health concern, with recent studies highlighting its persistent prevalence in diarrheal cases worldwide. In Beijing, during 2022–2023, ETEC was reported as the second most prevalent pathogen in patients with acute diarrhea [71]. Similarly, a study conducted in Ethiopia during 2021–2022 found that, ETEC accounted for 53.8 % of the 39 *E. coli* isolates carrying virulence genes, surpassing other diarrheagenic *E. coli* pathotypes in prevalence [72]. These epidemiological findings underscore the importance of understanding the genetic diversity of ETEC strains, particularly in context with ABR. ETEC clonal lineages with conserved multi-locus sequence types (MLSTs) and specific toxin/CF profiles often also display conserved O-antigens (Table 1). For instance, O169:H41/ ETEC ST182 has been identified as a frequent cause of foodborne outbreaks of diarrhea in USA, Japan and Korea [[73], [74], [75]] and ETEC ST182 was also found in airline waste at a German international airport [20]. These O169:H41/ST182 isolates belong to the major ETEC lineage L7 (Table 1) [12]**,** and increasing levels of resistance were identified in these isolates, i.e., the study from 2021 on airline waste found gene casettes including the ESBL *bla*_CTX-M-15_ and *qnr* in addition to many other ARGs [20].

**Table 1:** Characteristics of ten common ETEC lineages that are repeatedly isolated from patients with severe diarrhea globally. Data modified from von Mentzer et al., 2014 [12].

Lineage	Enterotoxin	Colonization factor	MLST	O antigen
L1	LT STh	CS1 + CS3	ST2353, ST4	O6
L2	LT STh	CS2 + CS3	ST4, ST48	O6
L3	LT STh, STh	CFA/I, CS7	ST173, ST	O78,O114, O126
L4	LT, STh	CS6, CS6 + CS8, CS21	ST1312	O25
L5	LT STh	CS5 + CS6	ST443	O115, O157
L6	STh	CFA/I	ST2332	ON3
L7	STp	CS6	ST182	O169:H41
L8	STh, STp	CS6	ST94	O148
L9	STp	C6	ST389	O27
L10	LT, STp	CS19	ST2368	O114

Genomic studies revealed that L7 ST182 ETEC contains a phage-like HMC2/SSU plasmid designated pAvM_E1373_29 [25] (Table 2), with conserved homology to plasmids found in other *E. coli*. These include an ETEC O169:H41 isolate F8111-1SC3 isolated in the 1970s [76], several *bla*_CTX-M-15_ positive phage-like HMC2/SSU plasmids e.g., pANCO1, pANCO2 from *E. coli* [77], a *bla*_CTX-M-15_ plasmid found by the authors of this review in *E. coli* ST648 from wastewater [30], and a ST131 isolate SC367ECC [78]. Hence, this plasmid that is stably integrated in ETEC lineage L7 is both able to pick up new ARGs and able to spread between different pathogenic *E. coli*. These results indicate that ESBL and multi-drug resistance might accumulate in the ETEC lineage L7 ST182 O169:H41 that carries the STp toxin and colonization factor CS6 and is frequently detected as a cause of foodborne outbreaks.

**Table 2:** Plasmids in ETEC lineages with ARGs.

MLST	Plasmids	Antibiotic resistance plasmid	ARGs	Reference
L1 (ST2353)	IncFII FII + FIB I1 FII	–	–	[25] [89]
L2 (ST4)	FII Y FII + FII	Y	*bla*_CTX–M–15_	[18] [25] [88]
L3 (ST173)	FII + FIB B/O/K/Z	B/O/K/Z	*TetAR,*	[25]
L3 (ST 5305)	IncFII I1 I1-like	FII	*sul2, tetB, dfrA8 strAB bla*_TEM-1B_*, ampC*	[24] [25]
L4 (ST1312)	IncFII, Inc FII + FIB	FII + FIB	*aadA1, tetR, tetA, sul1,* and *dfrh1 +* CS21 operon *bla*_CTX–M–27,_	[25]
L4 (ST1491)	FII FIB IncBOKZ		*bla*_CTX–M–15,_ qnrS1	[88] [89]
L5 (ST443)	FII FII	–	–	[24]
L6 (ST2332)	FII I1 B/O/K/Z	IncFII	*bla*_TEM-1B_*, tetAR, merRTPCADE*	[25]
L7 (ST182)	FII + FIB, FII	ND	*aadA5, mphA qnrS1, sul1 dfrA17 qacEΔ1 bla*_CTX–M–15,_	[20]
ST2040	B/O/K/Z	IncBOKZ	*bla*_CTX–M–15,_ qnrS1	[88] [90]
ST48	ND	ND	*bla*_TEM-1B_	[89]

ND Not determined.

Isolates belonging to lineage L4 (Table 1 and Table 2), which carries two large plasmids - one conjugative and one harboring the LT toxin genes [25] - are at risk of developing multi-drug resistance. The conjugative plasmid, pAvM_E1441_17, carries both the CF CS21 operon (conferring adhesion and biofilm capacity) and *aadA1, tetR, tetA, sul1,* and *dfrh1* ARGs indicating that this is a plasmid that can transfer both virulence and ABR traits while the LT gene in this isolate is located on another non-conjugative plasmid **[****25****]**. Re-analysis of historical data revealed that *tetAR* and *sul1* resistance was described to be mobile by conjugation in ETEC O25:H16 isolates from the 1970s while LT toxin genes were not mobilized in this study indicating location on a different plasmid that was not co-transferred nor mobilized by other means [79]. This strongly indicates that the old study [79] was performed on L4 O25:H16 isolates that had the same plasmid content 50 years ago as we see in isolates from recent patient samples [25]. It also shows that ETEC L4 might be able to transfer its ARGs and CS21 plasmid to other members of *E. coli* or Enterobacterales conferring both virulence and resistance at the same time. L4 (ST1312, O25) expresses LT STh and CS6. Hence CS6 positive ETEC isolates should be screened for ABR to study if additional ARGs are inserted into the conjugative plasmid of L4 ETEC.

In order to obtain a more updated view on ETEC isolates recovered from diarrheal samples and environmental water sources during the last decade, recent publications were screened for information of CF and toxin types, MLST-types, O-antigens, and/or available WGS data in relation to multi-drug resistance. Not all studies combined phenotypic with genotypic or WGS analysis and it should be noted that these methods do not always match.

In a study that examined ETEC prevalence in children aged less than 36 months in Zambia, the majority of the identified ETEC cases were LT STh/CS2 + CS3 and ST-toxin/CS6 [80]. Although resistance and MLST types were not studied in Sukwa et al., [80] ETEC with these toxin and CF profiles are reported to be multi-drug resistant and/or ESBL-positive in other studies [18,20]. It is likely that the Zambian isolates belong to lineages L2 (LT STh/CS2+ CS3+/-CS21) and L4 or L7, L8 or L9, all latter expressing CS6 in combination with ST-toxin (Table 1).

A study of 265 ETEC from clinics is Nepal collected between 2001 and 2016 found 40 ESBL isolates from 2008, 2013, 2014, and 2016. It was determined that 45.5 % (118/265) of the ETEC isolates were resistant to more than two antibiotics including ampicillin (AMP), trimethoprim/sulfamethoxazole (TMP-SMX), tetracyclin (TET) and CIP [18]. The primary ESBL gene identified was *bla*_CTX-M-15_ (80 %). The same study found that the CF combinations CS2 + CS3 + CS21 and CS6 + CS21 were significantly higher in the ESBL-positive ETEC population when compared to the ESBL-negative ETEC population. LT  STh/CS2 + CS3 + CS21 belongs to ETEC lineage 2 [12], which carry several plasmids including a IncY plasmid that was found to carry ARGs using long-read PacBio analysis of representative ETEC lineage isolates [25] (Table 2). The L2 IncY plasmid is a P1 phage-like plasmid (pAvM_1649_9) that is similar to p1107–99 K, and pEC2_5 isolated from human urine and p2448–3 from an UPEC ST131 strain isolated from blood, indicating that this plasmid can transfer between different pathogenic hosts. Hence, ST4 ETEC isolates belonging to L2 expressing LT   STh/CS2 + CS3 might be emerging multi-drug resistant clones (Table 2). ST4 isolates carrying ETEC toxins has been identified in surface water in Mexico [57] indicating that L2 isolates are also found in water sources. In addution L6 ETEC MLST2332 O128:H45 with multi-drug antibiotic resistance including CTX was identified in outbreaks of infantile diarrhea in neonate units in China in 2012 [81]. These isolates belong to Lineage 6 with toxin/CF profile STh/CFA/I ST2332 indicating early acquisition of ESBL genes in this ETEC lineage.

## 7 ESBL-ETEC are more common in South East Asia/India than in other continents

A study in Ethiopia conducted in 2021–2022 in children with diarrhea [72] found that ETEC isolates were resistant to CIP, AMP, amoxicillin (AMX), gentamicin (GEN), streptomycin (STR), trimethoprim (TMP), and/or TMP-SMX, but none of the isolates were resistant to CTX indicating lower prevalence of ESBL-ETEC in African ETEC isolates.

Corroborating these results Kantele and Lääveri, 2021 [16] conducted studies on Finnish travelers and found ESBL-diarrheal *E. coli* to be more associated with traveling to Asia while none of the diarrheagenic *E. coli* collected from travelers to Africa or Latin America were ESBL positive. Similar results with higher resistance in ETEC from South East Asia/India compared to Africa and Latin America were reported in a study of travelers from Spain 2011–2017 where ESBL genes were found in ST types ST23, ST38, ST131, ST1284, and ST5584 [23].The same study reported that resistance to TMP-SMX and Nalidixic acid (NAL) is high in African travelers with ETEC diarrhea and reported the first case of cephamycinase ACT-20 in an ETEC strain causing TD in a patient who had traveled to Central America.

## 8 ETEC MLST types not associated with ESBL carriage

Interestingly, a study in Nepal [18], found significant association between CS1 + CS3 positive isolates and the absence of ESBL. This could infer that Lineage L1 LT STh/CS1 + CS3 belonging to ST type 2353 or ST4 (Table 1 and Table 2) is not prone to integrate ESBL genes or other antibiotic resistance genes in general while L2 and CS6-positive lineages L4, L7, L8 and L9 do. L1 and L2 are very similar genetically and derive from a common ancestor [12], but the plasmid contents are different, indicating that it is the plasmidome that determines ARG profiles. In addition, to the authors knowledge multi-drug resistance has not yet been described in L5 LT STh/CS5 + CS6, ST443 (Table 2).

One study suggests that the presence of virulence genes and ARGs on the same plasmid favored specific virulent ETEC strains to keep ARGs [82]. However, in other studies of ETEC a negative correlation of ARGs and presence of ST-toxin was found in *E. coli* isolated from river surface water [83] and a negative correlation of the presence of LT and sulfamethoxazole (SMX) resistance has also been reported [84]. Results from the authors' previous studies indicate that in the most common clonal lineages of ETEC, ARGs are typically found on separate plasmids from those carrying virulence genes [25,27].

## 9 Are novel ESBL ETEC lineages emerging?

The emergence and rapid global spread of novel multi-drug resistant bacterial clones is well-documented [29,85]**.** We hypothesize that such clones arise from an optimal combination of the bacterial genome and acquired mobile elements, creating hypervirulent bacteria with enhanced abilities to disseminate in the environment, colonize hosts, cause disease and avoid antibiotic treatment.

*E. coli* ST410 is repeatedly found carrying plasmids with ARGs and is described as an emerging multi-drug resistant clone [37]. Recently, ST410 was found to carry plasmids with virulence factors for ETEC, including the colonization factor CS23 [86]. ST410 isolates carrying ETEC toxins have been identified in both water and patients in Bolivia and since their discovery the authors of this review have found evidence of global spread of isolates carrying the understudied colonization factor CS23 [87]. Given ST410's multi-drug resistant nature, a new type of virulent and resistant ETEC might be increasing in prevalence. ST410 has been isolated from water, companion animals, poultry, and humans [57], indicating its potential to persist across various hosts and environments.

A study in Korea [88] identified ETEC from patients with foodborne diarrhea between 2014 and 2018. Out of 126 ETEC isolates, 44 were ESBL carriers, with 32 showing multi-drug resistance to AMP, cefalotin (CEF), cefazolin (CFZ), CTX, and AXO. The predominant MLSTs identified were ST2040 and ST1491, both carrying *bla*_CTX-M-15_ and *qnrS*, and ST4 carrying *bla*_CTX-M-27_. These MLSTs belong to ETEC in phylogroup A [12]. They are part of clonal complex CC10, which includes various MLSTs such as ST10, ST4 (possible ETEC L2), ST1491 (belonging to L4) [89], and ST2040 [88] and ST1312 (L4). A study on ESBL Enterobacteriales in pre-washed salad identified ETEC ST2040 with *bla*_CTX-M-15_ on an IncB/O/K/Z plasmid and virulence profile ST/CFA/I [90]. Hence ETEC ST2040 might be a new ETEC variant associated with water- and foodborne diarrhea.

A study of traveler's diarrhea found that ETEC were resistant to multiple antibiotics, including AMP (48.9 %), amoxicillin-clavulanate (AMC) (7 %), CTX (14 %), TMP-SMX (44.2 %), chloramphenicol (CHL) (11.6 %), TET (39.5 %), nalidixic acid (NA) (44.2 %), CIP (21 %), and AZM (14 %). CTX resistance was only found in isolates from South east Asia/India. This study also identified two ETEC isolates belonging to ST131 carrying *bla*_*CTX-M-27*_, as well as ETEC isolates with *bla*_*CTX-M-15*_ in ST23, ST1284, and ST5584 [23]. These MLST types might also represent novel emerging variants of ESBL-ETEC and their global dissemination and virulence, particulary if ST131 can express ETEC toxins, remains to be confirmed.

An ETEC isolate was recovered by the authors from a Swedish WWTP in Stockholm in 2022. ETEC presence in Swedish wastewater has not previously been documented, to the authors' knowledge, however, a few outbreaks have previously been reported in Nordic countries [91,92]. ETEC are well adapted to survive and persist in aquatic environments and its presence in WWTPs therefore increases the risk of ETEC entering the environment through outlet water and potentially threatening human health. The Stockholm WWTP isolate belonged to MLST 6872 and was STh/CFA/I+CS21 positive and multi-drug resistant including ARGs *strA, strB, sul2, bla*_CTX-M-15_*, qnrS1* and *dfrA8_1*. MLST 6872 only differs in one of the seven MLST genes (Achtman scheme) compared to ST2332 (i.e L6 STh /CFA/I) indicating that the ETEC isolate has evolved from the clonal L6 lineage. A search in Enterobase found several additional isolates recovered worldwide and although not annotated as ETEC, they carried ST-toxin genes upon manual inspection. The Swedish ETEC isolate contained IncF and IncI plasmids, where the IncI plasmid containing ARGs *bla*_CTX-M-15_ and *qnrS1* was shown to be conjugative. The IncI plasmid has been found across the globe [93], highlighting its conjugative ability of spreading quickly both inter- and intra-species as well as highlighting the potential for widespread ETEC multi-drug resistance. Hence L6 ETEC and its derivatives might also evolve into multi-drug resistance and ESBL-carriage.

## 10 Conclusion

The transmission of ABR ETEC in aquatic environments, hosts, and vectors is closely interconnected, with genetically identical clones detectable across these domains. The frequent detection of ETEC in wastewater, rivers, lakes, and irrigation water highlights the critical role of aquatic environments as reservoirs and vectors for these pathogens. The ability of ETEC to thrive in diverse and often harsh conditions indicates that ABR ETEC and their clones have developed adaptive mechanisms that enhance their survival and transmission.

The emergence of novel multi-drug resistant ETEC clones, such as those carrying the CTX-M-15 and CTX-M-27 encoding ARGs, underscores the urgent need to understand how virulence and resistance plasmids facilitate ETEC dissemination. The present review has, through comparative analyses of several studies, identified several ETEC clonal lineages which might be emerging ESBL positive and multi-drug resistant ETEC lineages. Specifically, CS6 positive ETEC belonging to ST182, CS2 + CS3 positive ETEC belonging to ST4 and CFA/I positive ETEC belonging to ST2332. The prevalence of ABR ETEC in treated effluents from hospital and urban wastewater treatment plants suggests that these environments are hotspots for the spread of ABR pathogens to the environment. This issue is particularly severe in countries with high rates of diarrheal diseases, where inadequate sanitation and fecal pollution exacerbate the problem. To address the threat posed by these emerging pandemic clones, it is essential to investigate the genetic and environmental factors that enable the acquisition and persistence of virulence and resistance plasmids in ETEC. Interestingly, we have identified putative novel ETEC clones that might be emerging multi-drug resistant ESBL clones, including a Swedish ESBL STh/CFA/I + CS21 isolate that has been isolated in other studies and deposited in Enterobase but not previously identified as ETEC. While the presence of ETEC isolates until now has remained underdetected in e.g., WWTP studies, a recent update of VirulenceFinder will aid in identification of novel ETEC clones globally [87]. By understanding how and why novel clones emerge, we can develop strategies to control the spread of AMR *E. coli* and ETEC in aquatic environments, protect public health, and prevent the emergence of new, more virulent, resistant strains. Comprehensive surveillance and molecular studies including detailed WGS metadata are therefore vital for identifying and monitoring these evolving pathogens and implementing effective interventions to stop their dissemination.

## Funding

ÅS, EJ, and AJMR would like to thank the EU and 10.13039/501100004359Swedish Research Council for funding in the frame of the collaborative international consortium PARRTAE financed under the ERA-NET Aquapollutants Joint Transnational Call (GA n^a^869178). This ERA-NET is an integral part of the activities developed by the Water, Oceans and AMR Joint Programming activities. AvM acknowledges support from the 10.13039/501100005761Sahlgrenska Academy International Starting Grant, and the 10.13039/501100004359Swedish Research Council for funding grant no 2022–01449. AJM-R acknowledges the 10.13039/501100001862Swedish Research Council for Environment, Agricultural Sciences and Spatial Planning (FORMAS) for funding, grant ref. 2023–01097. ÅS would like to thank the 10.13039/501100004359Swedish Research Council for funding of grants no 2019–04202, 2020–01941, and 2023–03028.

## CRediT authorship contribution statement

**Enrique Joffré:** Writing – review & editing, Visualization. **Alberto J. Martín-Rodríguez:** Writing – review & editing. **Annie Justh de Neczpal:** Writing – review & editing. **Astrid von Mentzer:** Writing – review & editing. **Åsa Sjöling:** Writing – review & editing, Writing – original draft, Funding acquisition, Conceptualization.

## Declaration of competing interest

The authors declare that they have no known competing financial interests or personal relationships that could have appeared to influence the work reported in this paper.

## Data Availability

Data will be made available on request.
